# GP73-mediated secretion of AFP and GP73 promotes proliferation and metastasis of hepatocellular carcinoma cells

**DOI:** 10.1038/s41389-021-00358-3

**Published:** 2021-10-14

**Authors:** Yujuan Liu, Jiyin Wang, Ruixiang Yang, Yuning Cheng, Yue Zhou, Hui Li, Wei Jiang, Xiaowei Zhang

**Affiliations:** Department of Biochemistry and Biophysics, School of Basic Medical Sciences, Beijing Key Laboratory of Protein Posttranslational Modifications and Cell Function, Peking University Health Science Center, Beijing, PR China

**Keywords:** Cancer screening, Cell migration

## Abstract

Golgi protein 73 (GP73) and alpha fetoprotein (AFP) serve as biomarkers for the diagnosis of hepatocellular carcinoma (HCC), and their serum levels correlate with patients’ outcomes. However, the mechanisms underlying these correlations are unknown. Here we show that GP73 increased the secretion of AFP through direct binding to AFP, thereby promoting the proliferation and metastasis of HCC cells that expressed AFP and its receptor (AFPR). Extracellular GP73 contributed to the proliferation and metastasis of HCC cells independent of AFP and AFPR. Moreover, extracellular AFP and GP73 synergized to enhance the malignant phenotype of HCC cells. Furthermore, extracellular GP73 and AFP inhibited the antitumor effects of sorafenib and synergistically increased the drug resistance of HCC cells. These findings, which reveal the mechanism of GP73-mediated secretion of AFP and its effects on the malignant phenotype of HCC cells, provide a comprehensive theoretical basis for the diagnosis and treatment of HCC and identify potential drug targets.

## Introduction

Hepatocellular carcinoma (HCC) is the sixth most frequent cancer worldwide, accounting for approximately new 841,000 cases and 782,000 deaths each year [1]. The main risk factors for HCC are chronic infection with hepatitis B virus (HBV) or hepatitis C virus (HCV), aflatoxin‐contaminated food, and heavy alcohol consumption [2, 3]. As most HCCs are diagnosed at an advanced stage, and the efficacies of current treatments are insufficient, consequently mortality and recurrence rates are high [4, 5]. Therefore, early diagnosis and more effective treatments are required for the prevention and treatment of HCC. Thus, new highly sensitive and specific markers for HCC are critically important for these purposes, particularly those that can be easily and noninvasively measured, such as molecules present in sera.

The serum levels of the glycoprotein alpha fetoprotein (AFP) are widely used for diagnosis of HCC, as well as for screening, determining efficacy, and detecting recurrence [6]. Recent studies show that AFP acts as an oncoprotein that contributes to the progression of HCC [7], and intracellular AFP acts as a signaling molecule that mediates multiple cellular processes. The interaction between AFP and PTEN inhibits the function of latter, leading to malignant proliferation of HCC cells through the activation of the PI3K/AKT signaling pathway [8]. Similarly, the interaction between AFP and caspase-3 inhibits apoptosis of HCC cells by blocking the caspase signaling cascade [9, 10]. Furthermore, AFP blocks the formation of complexes between retinoic acid and its receptor, which decrease the expression of GADD153, GADD45A, and Fn14, thereby promoting the abnormal growth of HCC cells [11–13]. AFP promotes the migration of HCC cells and invasion through upregulating the expression of metastasis-related genes such as *K19*, *EpCAM*, *MMP2*, *MMP9*, and *CXCR4* [14, 15]. Binding of extracellular AFP to its receptor (AFPR) activates the Ca^2+^ and cyclic adenosine 3’,5’-monophosphate (cAMP) signaling pathways, thereby promoting the proliferation and metastasis of HCC cells [16, 17]. Overall, AFP serves as a serum marker for the diagnosis of HCC and plays an important role in the progression of HCC. However, AFP levels may be normal in as many as 20% of patients with HCC, particularly during its early stages. Therefore, identification of sensitive and specific serum biomarkers for the early detection of HCC is urgently required.

Golgi protein 73 (GP73) (also called GOLM1 or GOLPH2) is a type II Golgi-localized integral membrane protein that is predominantly expressed by cells of the epithelial lineage, although at low levels in hepatocytes in normal liver. The expression of GP73 significantly increases in virus (HBV, HCV)-infected liver [18]. GP73 serves as a potential serum marker for HCC and is highly expressed in several types of tumor cells [19, 20]. For example, a study focused on the molecular mechanisms of GP73 that promote HCC progression and metastasis showed that GP73 drives metastasis through its interaction with EGFR to regulate its cell-surface recycling to promote the epithelial–mesenchymal transition in HCC cells [21]. Moreover, GP73 interacts with MMP2 or MMP7 in HCC cells to promote their transportation and secretion, thereby promoting metastasis of HCC cells [22, 23]. Together, these findings support the conclusion that GP73 is involved in the development of HCC via multiple mechanisms. Another study suggests that assays of GP73 achieve greater sensitivity and specificity than those for AFP, and GP73 serum levels increase with the malignant potential of liver diseases such as hepatitis, hepatic cirrhosis, and HCC [24]. Although the mechanisms that lead to elevated GP73 serum levels are unknown, their roles in its secretion and potential role in the diagnosis of HCC are of great interest.

Here we show a coordinated relationship between GP73 and AFP. Thus, high levels of GP73 mediated the secretion of AFP as well as itself, and of the former through intracellular secretion via direct interaction with AFP, which promotes the malignant phenotype of HCC cells. Furthermore, extracellularly secreted GP73 significantly contributes to the proliferation and metastasis of HCC cells that do not express AFP. Moreover, extracellular GP73 and AFP synergize to increase the malignancy of HCC cells and resist the antitumor effects of sorafenib. These findings illuminate new approaches for diagnosing and treating HCC.

## Results

### GP73 promotes AFP secretion

**Fig. 1:**
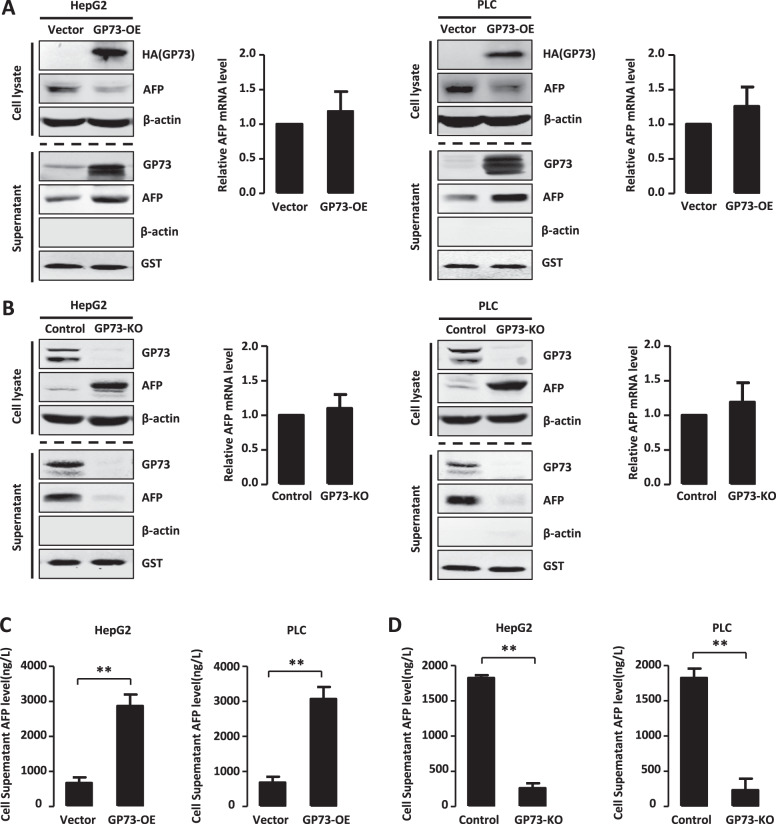
GP73 promotes AFP secretion. **A** GP73 overexpression decreases the protein level of intracellular AFP and increases the level of extracellular AFP. The protein levels of GP73 and AFP in cell lysate and supernatant were measured by western blotting in HepG2 and PLC cells transfected with PCDNA3-GP73 or PCDNA3 (as a control). β-actin or GST was respectively used as an intracellular or extracellular loading control. The mRNA level of AFP in cell lysate was measured by Real-time PCR. **B** GP73 knockout increases the protein level of intracellular AFP and decreases the level of extracellular AFP. The protein levels of GP73 and AFP in cell lysate and supernatant were measured by western blotting in HepG2 and PLC cells mediated GP73 knockout by CRISPR/Cas9 system. β-actin or GST was respectively used as an intracellular or extracellular loading control. The mRNA level of AFP in cell lysate was measured by Real-time PCR. **C** GP73 overexpression increases the secretion level of AFP. Stable transfected HepG2 and PLC cells overexpressed GP73. Cell culture supernatant was collected and AFP level was measured by ELISA. **D** GP73 knockout decreases the secretion level of AFP. The expression of GP73 in HepG2 and PLC cells were knockout by CRISPR/Cas9 system. Cell culture supernatant was collected and AFP level was measured by ELISA. Error bars represent S.D. *p < 0.05, **p < 0.01.

Clinical data show that the serum levels of GP73 and AFP are consistent in patients with HCC [25]. Thus, we investigated whether GP73 influenced AFP secretion by HCC cells. For this purpose, we transfected HepG2 and PLC cells with the expression vector HA-GP73 and detected intracellular AFP expression using western blotting and real-time PCR. GP73 overexpression (GP73-OE) decreased AFP levels in HepG2 and PLC cells but did not alter AFP mRNA levels (Fig. 1A, top panels). To further address the functions of GP73, we determined the levels of secreted GP73 and AFP and found the secretion level of AFP was also elevated with the secretion level of GP73 increased (Fig. 1A, bottom panels).

To analyze the regulation of endogenous AFP levels by GP73, we constructed GP73-knockout cells using the CRISPR/Cas9 system. The sgRNA targeted the third exon of the GP73 gene to prevent its transcription. GP73 knockout (GP73-KO) upregulated AFP levels in the cytoplasm but had no effect on AFP mRNA levels in each cell line. Conversely, the levels of secreted GP73 and AFP were reduced in two GP73-knockout cell lines (Fig. 1B, bottom panels). To directly detect the levels of secreted AFP, we collected cell culture supernatants and measured AFP levels using an ELISA. We found that the levels of secreted AFP increased in cells overexpressing GP73 but were reduced in GP73-knockout cells (Fig. 1C, D). These results indicated that GP73 mediates AFP secretion, GP73 overexpression promotes its secretion as well as that of AFP, and GP73 knockout inhibits secretion.

### GP73 interacts with AFP in vivo and in vitro

**Fig. 2:**
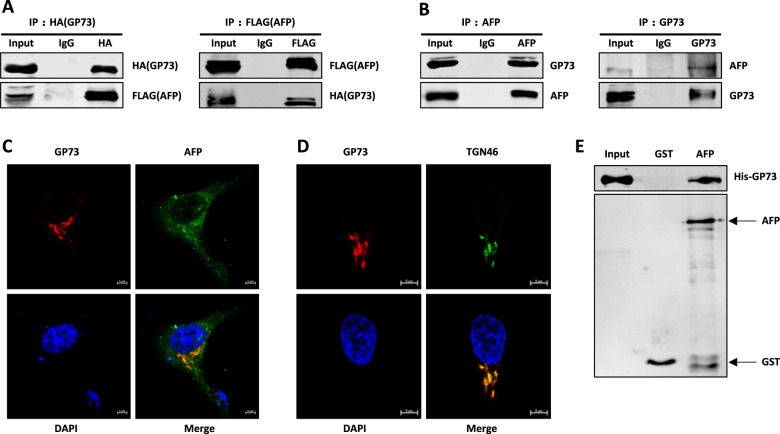
AFP interacts with GP73 in vivo and in vitro. **A**, **B** GP73 interacts with AFP in vivo. **A** HEK293T cells co-transfected with FLAG-AFP and HA-GP73 were lysed with IP lysis buffer and subjected to immunoprecipitation with anti-HA or anti-FLAG antibodies followed by western blotting with anti-HA or anti-FLAG antibodies. **B** HepG2 cells were lysed with IP lysis buffer and subjected to immunoprecipitation with anti-AFP or anti-GP73 antibodies followed by western blotting with anti-AFP or anti-GP73 antibodies. **C** GP73 co-localizes with AFP in cytoplasm. HepG2 cells was stained with GP73 antibody (red) and AFP antibody (green). DAPI (blue), nucleus. Scale bar, 5 µm. **D** GP73 localizes in the Golgi apparatus. HepG2 cells was stained with GP73 antibody (red) and TGN46 (green). DAPI (blue), nucleus. Scale bar, 5 µm. **E** GP73 interacts with AFP in vitro. GST pull-down assay was performed by using His-GP73 and GST or GST-AFP proteins in vitro and analyzed by western blotting with anti-His antibody.

We next investigated whether GP73 binds to AFP in HEK293T cells co-transfected with HA-GP73 and FLAG-AFP expression vectors. The cells were subjected to immunoprecipitation and western blotting using anti-FLAG or anti-HA antibodies. As shown in Fig. 2A, exogenous GP73 bound to exogenous AFP in vivo. The interaction of endogenous GP73 with AFP in HepG2 cells was further assessed using coimmunoprecipitation assays employing anti-AFP or anti-GP73 antibody followed by western blotting. Similar to the exogenous proteins, endogenous GP73 bound to endogenous AFP (Fig. 2B). Using anti-AFP or anti-GP73 antibody, we performed immunofluorescence staining with HepG2 cells to test the co-localization between endogenous GP73 and AFP. The result verified that endogenous GP73 co-localizes with AFP in the cytoplasm (Fig. 2C). Since GP73 is a Golgi-localized protein, we used TGN46, which is Golgi apparatus-specific antibody, to stain Golgi apparatus (green). GP73 was stained with GP73 antibody (red). As shown in Fig. 2D, the immunofluorescence merge images (yellow) showed that GP73 localized in the Golgi. Together with Fig. 2C, we can conclude that GP73 co-localizes with AFP in the Golgi apparatus in cytoplasm. To determine whether GP73 and AFP directly interacted, we performed a GST-AFP pull-down assay. The result showed that GST-AFP, but not GST, bound His-GP73 (Fig. 2E). These data suggest that GP73 physically interacts with AFP in vivo and in vitro.

### GP73-mediated AFP secretion depends on the interaction of GP73 with AFP

**Fig. 3:**
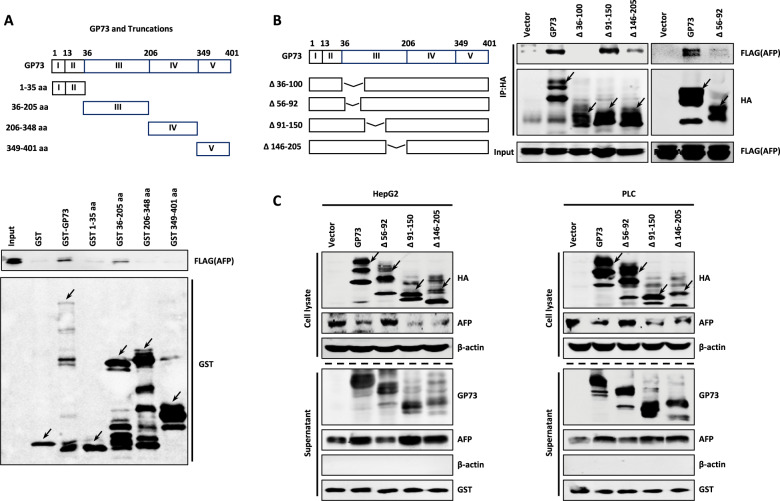
GP73-mediated AFP secretion depends on the interaction of GP73 with AFP. **A** AFP interacts with domain III of GP73. GST pull-down assay was performed by using lysate of HEK293T cells transfected with FLAG-AFP and GST or GST-tagged various domain of GP73 proteins and analyzed by western blotting with anti-FLAG antibody. **B** AFP interacts with 56–92 aa in domain III of GP73. HEK293T cells co-transfected with FLAG-AFP and HA-tagged GP73, Δ36–100, Δ56–92, Δ91–150, or Δ146–205 were lysed with IP lysis buffer and subjected to immunoprecipitation with anti-HA or anti-FLAG antibodies followed by western blotting with anti-HA or anti-FLAG antibodies. **C** Deletion of 56–92 aa in domain III of GP73 reduces AFP secretion. The protein levels of GP73 and AFP in cell lysate and supernatant were measured by western blotting in HepG2 and PLC cells transfected with vector, GP73, Δ56–92, Δ91–150, or Δ146–205 plasmid. β-actin or GST was respectively used as an intracellular or extracellular control.

To determine whether the interaction of GP73 with AFP affects the secretion of AFP, we first ascertained the domains of GP73 required for their interaction using a set of GST-tagged GP73 deletion mutants (Fig. 3A, top panel) in a GST pull-down assays (Fig. 3A, bottom panel). As shown in Fig. 3A, domain III of GP73 (amino acid residues 36–205) directly interacted with AFP, whereas its other domain did not. To determine a more specific region required for the interaction of GP73 with AFP, we constructed four deletion mutants of domain III (Fig. 3B, left panel) and used these mutants in coimmunoprecipitation assays (Fig. 3B, right panel). As shown in Fig. 3B, the two mutants Δ36–100 and Δ56–92 (deletion of amino acid residues 36–100 and 56-92 individually) did not bind AFP, whereas the other domains did, indicating that the region spanning amino acid residues 56–92 is required for the binding of GP73 to AFP.

Next, we individually transfected HepG2 and PLC cells with wild-type GP73 as well as with the GP73 truncated mutants Δ56–92, Δ91–150, or Δ146–205. We measured intracellular and extracellular AFP levels using western blotting. Overexpression of wild-type GP73, Δ56–92, Δ91–150, or Δ146–205 increased the secretion levels of itself (Fig. 3C). In contrast, wild-type GP73, Δ91–150, or Δ146–205 reduced the expression of intracellular AFP and increased the levels of secreted AFP, whereas Δ56–92 had no effect on the levels of intracellular and extracellular AFP. These results suggested that the increase in the levels of secreted AFP depends on the interaction of GP73 with AFP, specifically requiring amino acid residues 56–92 of GP73.

### GP73-mediated secretion promotes proliferation and metastasis of HCC cells

**Fig. 4:**
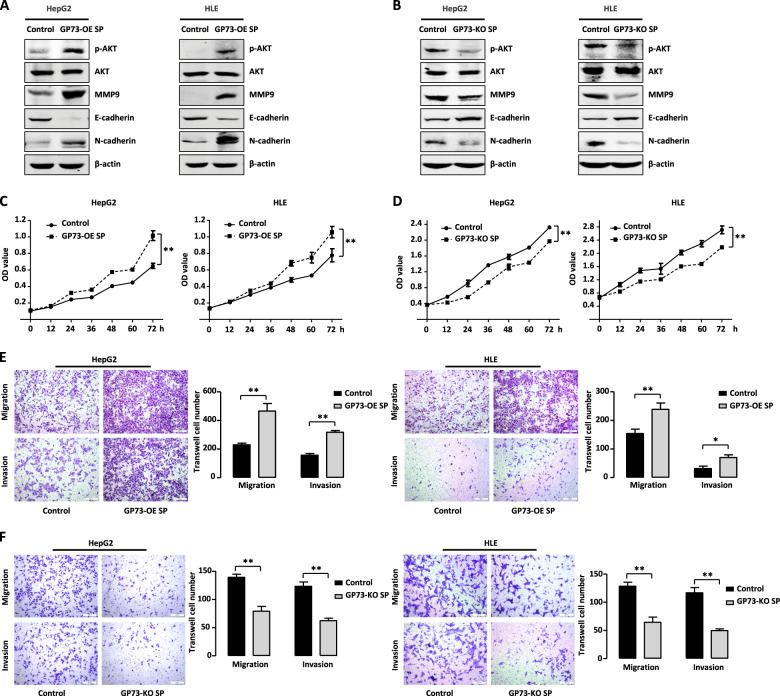
GP73-mediated secretion promotes proliferation and metastasis of HCC cells. **A**, **B** GP73-mediated secretion influences the expression of proliferation and metastasis-related proteins. **A** HepG2 and HLE cells treated with or without GP73 overexpression supernatant (GP73-OE SP) were analyzed by western blotting. **B** HepG2 and HLE cells treated with or without GP73 knockout supernatant (GP73-KO SP) were analyzed by western blotting. β-actin was used as a loading control. **C**, **E** GP73-OE SP treatment promotes proliferation, migration, and invasion of HCC cells. MTT assay (**C**) and Transwell assay (**E**) were conducted with HepG2 and HLE cells treated with or without GP73-OE SP. Scale bar, 200 μm. **D**, **F** GP73-KO SP treatment inhibits proliferation, migration and invasion of HCC cells. MTT assay (**D**) and Transwell assay (**F**) were conducted with HepG2 and HLE cells treated with or without GP73-KO SP. Scale bar, 200 μm. Error bars represent S.D. *p < 0.05, **p < 0.01.

To determine whether GP73-mediated secretion affects the proliferation and metastasis of HCC cells, we constructed HepG2 cells that stably overexpressed GP73. We collected cell culture supernatants (GP73-OE-SP) from these cells and then tested their effects on proteins involved in proliferation and metastasis expressed by HepG2 and HLE cells. As shown in Fig. 4A, GP73-OE-SP treatment increased the levels of phosphorylated AKT without affecting total AKT levels. Furthermore, such treatment upregulated the levels of MMP9 and N-cadherin and decreased those of E-cadherin. HepG2 and HLE cells treated with supernatants collected from cultures of GP73-knockout cells (GP73-KO-SP), reduced the levels of phosphorylated AKT, MMP9, and N-cadherin, and increased the level of E-cadherin (Fig. 4B). Thus, GP73-mediated secretion upregulated the expression in HCC cells of proteins that mediate proliferation and metastasis.

Next, we investigated the effects of GP73-mediated secretion on proliferation, migration, and invasiveness of HepG2 and HLE cells. As shown in Fig. 4C, GP73-OE-SP increased proliferation rate of both cell lines. In contrast, cells treated with GP73-KO-SP proliferated at a lower rate than control cells (Fig. 4D). Furthermore, the presence or absence of GP73-OE-SP had no effect on the growth of normal L02 liver cells (Fig. S1). We next performed migration and invasion assays of HepG2 and HLE cells treated with GP73-OE-SP or GP73-KO-SP. Consistent with the changes in the levels of metastasis-related proteins, GP73-OE-SP treatment promoted the migration and invasiveness of HCC cells (Fig. 4E), which decreased in the presence of GP73-KO-SP (Fig. 4F). Together, these findings indicated that GP73-mediated secretion contributes to the malignant phenotype of HCC cells through upregulating the expression of proteins involved in proliferation and metastasis but has no effect on the growth of normal liver cells.

### Secreted AFP and GP73 synergistically promote the proliferation and metastasis of HCC cells

**Fig. 5:**
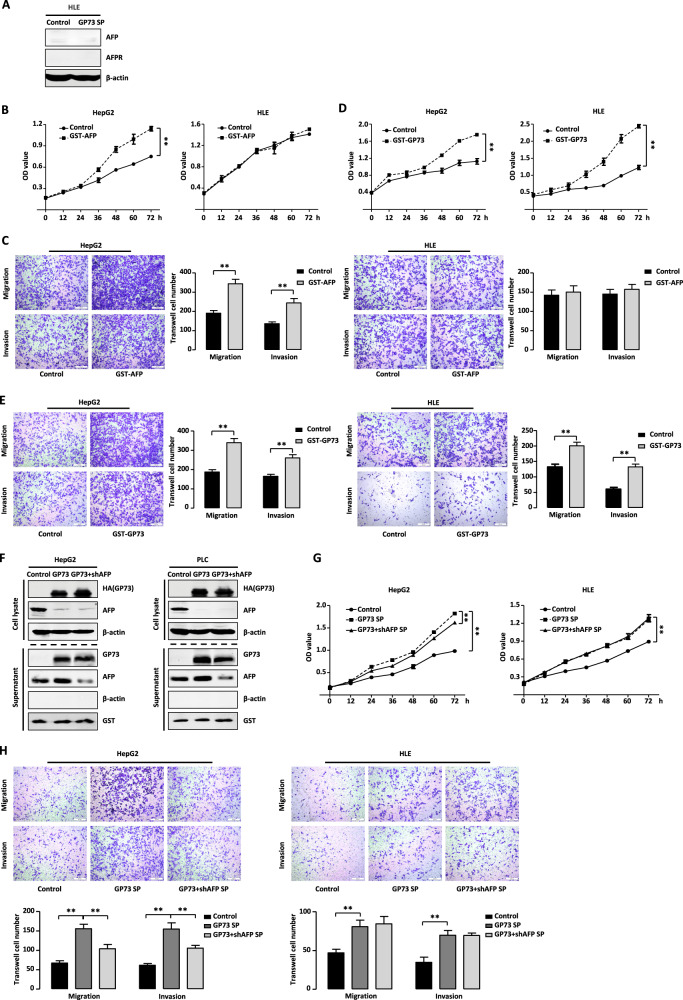
GP73-mediated secretion of AFP and GP73 promotes proliferation and metastasis of HCC cells synergistically. **A** HLE is AFP and AFPR negative cell line. The protein levels of AFP and AFPR in HLE cells treated with or without GP73 overexpression supernatent (GP73-OE SP) were analyzed by western blotting. β-actin was used as a control. **B**, **C** GST-AFP promotes cell proliferation, migration, and invasion only in HepG2 cells. MTT assay (**B**) and Transwell assay (**C**) were conducted with HepG2 and HLE cells with or without GST-AFP treatment. Scale bar, 200 μm. **D**, **E** GST-GP73 promotes cell proliferation, migration, and invasion both in HepG2 and HLE cells. MTT assay (**D**) and Transwell assay (**E**) were conducted with HepG2 and HLE cells with or without GST-AFP treatment. Scale bar, 200 μm. **F** AFP knockdown decreases GP73-mediated AFP secretion. The protein levels of GP73 and AFP in cell lysate and supernatant were measured by western blotting in HepG2 and PLC cells stably transfected with GP73 with or without AFP knockdown plasmid. β-actin or GST was respectively used as an intracellular or extracellular control. **G**, **H** AFP knockdown decreases cell proliferation, migration, and invasion of GP73-mediated secretion only in HepG2 cells. MTT assay (**G**) and Transwell assay (**H**) were conducted with HepG2 and HLE cells with treatment of cell culture supernatant in panel **F**. Scale bar, 200 μm. Error bars represent S.D. *p < 0.05, **p < 0.01.

Our present data demonstrate that GP73 facilitates the secretion of AFP. Li et al. reported that extracellular AFP binds to AFPR, thereby promoting the proliferation and metastasis of HCC cells [16]. Therefore, we assumed that the role of GP73-mediated secretion might depend on AFP secretion. Intriguingly, as shown in a previous study [26] and in Fig. 5A, HLE did not express AFP or AFPR in contrast to HepG2 cells. Thus, the effect of GP73-mediated secretion on HLE cells was independent of AFP and AFPR. We therefore asked how GP73-mediated secretion played a role in the proliferation and metastasis of HLE cells. Previous study showed that GP73 is a secretory protein that promotes the proliferation and metastasis of HCC cells through multiple intracellular signaling pathways [27]. Therefore, we assumed that secreted GP73 and AFP might possess these same activities.

To verify this assumption, we added GST-AFP to the culture medium of HepG2 and HLE cells and determined whether secretory AFP only affected HepG2 cells. As shown in Fig. 5B, the proliferation of HepG2 cells significantly increased in the presence of GST-AFP; however, the proliferation rates of HLE cells were comparable in the presence or absence of GST-AFP. Analysis of cell migration and invasion using Transwell assay showed that GST-AFP treatment enhanced the migration and invasiveness of HepG2 cells but not those of HLE cells (Fig. 5C). Furthermore, GST-GP73 exerted similar facilitating effects on HCC cells, supporting the importance of extracellular GP73 in the enhancement of the malignant phenotype of HCC cells. As shown in Fig. 5D, HepG2 and HLE cells proliferated at higher rates in the presence of GST-GP73, and GST-GP73 treatment increased the migration and invasiveness of both cell lines (Fig. 5E).

To determine whether GP73 binds to a specific receptor on the cell surface, we co-cultured FITC tagged-GP73 with HepG2 cells. As shown in Fig. S2, FITC tagged-GP73 localized outside the nucleus, and there were spaces between its signals and the nucleus, indicating that extracellular GP73 may bind to a receptor outside the nucleus. Thus, these results indicated that extracellular AFP or GP73 might enhance the malignant phenotype of HCC cells through receptors specific for each.

To strengthen evidence supporting the role of secretory AFP and GP73 in their contributions to GP73-mediated secretion from HCC cells, HepG2 and PLC cells were stably transfected with GP73 with or without lentivirus knockdown AFP. As shown in Fig. 5F, GP73 overexpression increased its own levels of secretion as well as those of AFP, while AFP knockdown reduced AFP secretion. Next, HepG2 and HLE cells were each individually treated with the three groups of cell culture supernatants harvested from the cells in Fig. 5F. AFP knockdown reduced the GP73-induced increase in the proliferation of HepG2 cell, but had no effect on HLE cells (Fig. 5G). Similarly, migration and invasiveness were decreased by treatment with the AFP knockdown supernatant only in HepG2 cells (Fig. 5H). Together, these findings indicated that GP73-mediated AFP and GP73 extracellular secretion synergistically facilitate the proliferation, migration, and invasiveness of HepG2 cells.

We collected cell culture supernatants from HepG2 cells that stably overexpressed GP73 (GP73-OE-SP). Using coimmunoprecipitation assay, we found that GP73 also bound to AFP in supernatant (Fig. S3). The data indicate that GP73-OE-SP promotes proliferation/migration signaling may be through GP73-AFP interaction. Together with Fig. 5B–G, GP73 or AFP individually also promotes proliferation/migration signaling in HCC cells. Thus we speculated that GP73-OE-SP promotes proliferation/migration signaling might depend on both GP73-AFP interaction and individual molecule effect.

### Extracellular secretion of GP73 is required to facilitate AFP secretion and maintain the malignant phenotype of HCC cells

**Fig. 6:**
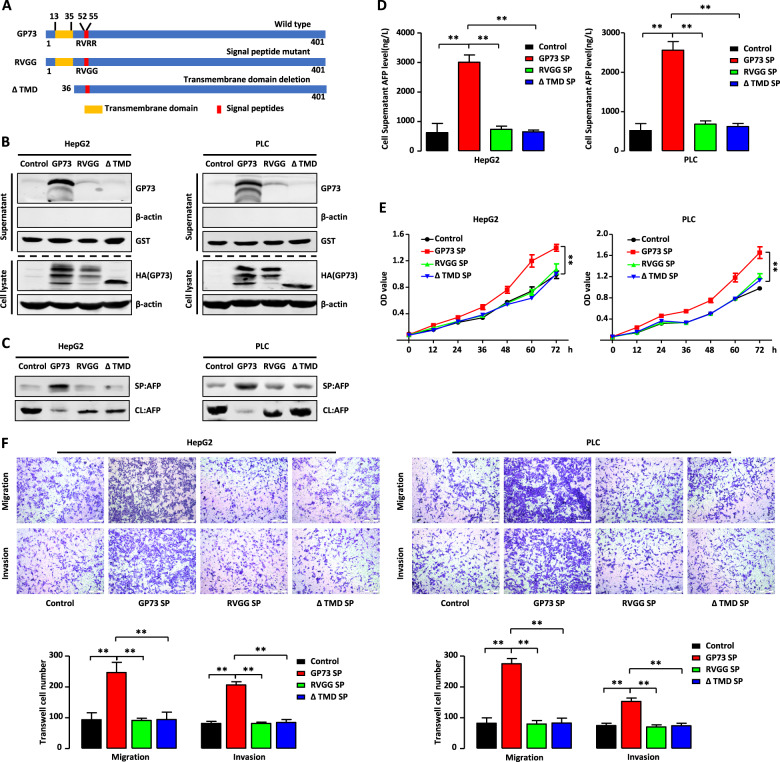
GP73 extracellular secretion is indispensable for facilitating AFP secretion and maintaining malignant phenotype of HCC cells. **A** Schematic diagram depicts GP73 wild type, signal peptide mutant (RVGG), and transmembrane domain deletion mutant (ΔTMD). **B** RVGG or ΔTMD mutant decreases GP73 extracellular secretion. The protein level of GP73 in cell lysate and supernatant in HepG2 and PLC cells transfected with vector, GP73, RVGG, or ΔTMD plasmid were measured by western blotting. β-actin or GST was respectively used as an intracellular or extracellular control. **C**, **D** RVGG or ΔTMD mutant decreases GP73-mediated AFP secretion level. **C** The protein level of AFP in cell lysate (CL) and supernatant (SP) in above cells were measured by western blotting. **D** The level of AFP in cell culture supernatant in above cells were measured by ELISA. **E**, **F** RVGG or ΔTMD mutant decreases cell proliferation, migration and invasion induced by GP73-mediated secretion. MTT assay **E** and Transwell assay **F** were conducted with HepG2 and PLC cells with treatment of cell culture supernatant in **B**. Scale bar, 200 μm. Error bars represent S.D. *p < 0.05, **p < 0.01.

GP73 is a Golgi-localized integral membrane protein with a signal peptide and transmembrane domains required for its localization to the Golgi membrane [27, 28]. We next investigated whether the domains were required for GP73-mediated secretion. As shown in Fig. 6A, we constructed two mutants. The first, RVGG, was mutated (RR to GG) in the region of the conserved protease cleavage site in the signal peptide, causing loss of extracellular secretion. The second, ΔTMD, harbors a deletion in the trans-Golgi membrane domain (amino acid residues 1–35 of the N-terminal domain), causing loss of its ability to localize to the Golgi. We individually transfected HepG2 and PLC cells with wild-type GP73, RVGG, or ΔTMD. As shown in Fig. 6B, the secretion of GP73 in HepG2 and PLC cells stably expressing RVGG or ΔTMD was significantly reduced compared with that of cells expressing wild-type GP73, indicating that the two domains of signal peptide (RVRR) and transmembrane domain (TMD) are required for GP73 secretion. The truncated mutants of GP73, Δ56–92, Δ91–150, and Δ146–205, contained the two domains of RVRR and TMD, which are indispensable for GP73 secretion. Therefore, overexpression of these truncated mutants increased the secretion levels themselves (Fig. 3C).

We then used western blotting and ELISA to determine the levels of secreted AFP. Similar to the levels of secreted GP73, those of secreted AFP were lower in HepG2 and PLC cells stably expressing the RVGG or ΔTMD mutant compared with cells expressing wild-type GP73 (Fig. 6C, D). The data indicate that the deficiency in extracellular secretion of GP73 decreases the level of secreted AFP.

We next asked whether the extracellular secretion of GP73 was functionally associated with the malignant phenotype of HCC cells. As shown in Fig. 6E, GP73-SP treatment significantly increased the proliferation rate of HepG2 and PLC cells, whereas the proliferation rates of cells treated with RVGG-SP or ΔTMD-SP were similar to those of the control cells. Furthermore, Fig. 6F shows that GP73-SP treatment increased the migration and invasiveness of HepG2 and PLC cells but RVGG-SP or ΔTMD-SP had no effect on HCC cells.

Together, these results indicated that intact signal peptide and transmembrane domain structures are required for extracellular secretion of GP73, which is indispensable for facilitating AFP secretion and maintaining the malignant phenotype of HCC cells.

In addition, we wondered whether the truncated mutants of GP73, Δ56–92, Δ91–150, and Δ146–205, affect GP73’s biological function since their overexpression could increase the secretion levels of itself? As shown in Fig. S4, GP73-SP, Δ91–150-SP or Δ146–205-SP increased proliferation (Fig. S4A), migration and invasiveness (Fig. S4B) of HepG2 cells, while Δ56–92-SP had no effect on cell growth (Fig. S4A) and metastasis (Fig. S4B). The data indicated that GP73-induced proliferation and metastasis of HCC cells requires AFP binding domain (amino acid residues 56–92) of GP73.

It was reported that mTOR up-regulates the expression of GP73, so GP73 level can be reduced by rapamycin, which is an inhibitor of mTOR [29]. Therefore, we used rapamycin to inhibit the function of GP73 and analyzed its binding to AFP. As shown in Fig. S5A, rapamycin treatment reduced GP73 binding to AFP. Furthermore, rapamycin treatment decreased the levels of intracellular and secreted GP73 (Fig. S5B). We analyzed the levels of proliferation- and metastasis-related proteins and the phenotypes of the cells. As shown in Fig. S5C, rapamycin significantly reduced the levels of phosphorylated AKT, MMP9, and N-cadherin and increased the level of E-cadherin. Accordingly, rapamycin significantly inhibited cell growth (Fig. S5D) and metastasis (Fig. S5E). Altogether, the results indicated that rapamycin inhibits malignancy of HCC cells through reducing the binding of GP73 and AFP.

### Extracellular GP73 and AFP attenuate the antitumor efficacy of sorafenib

**Fig. 7:**
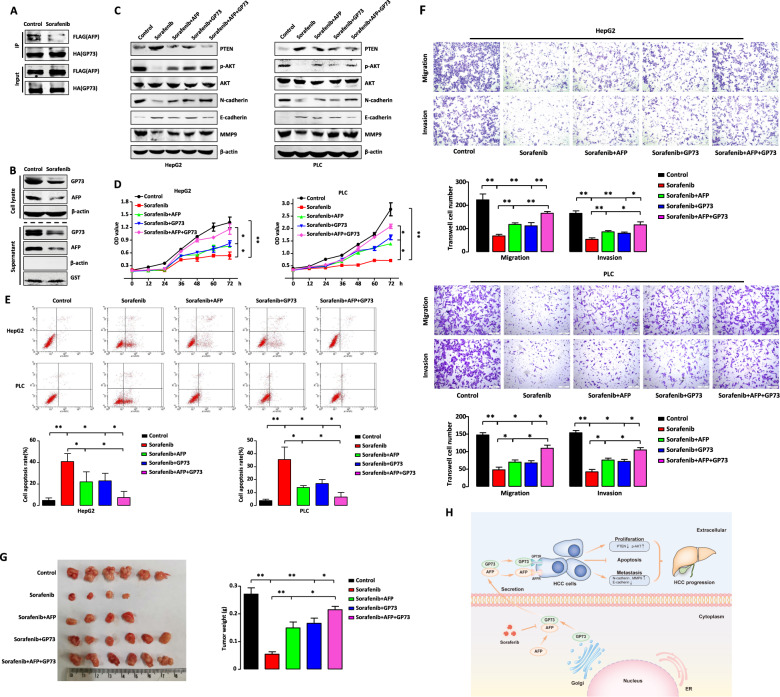
Extracellular GP73 and AFP attenuates sorafenib antitumor efficiency. **A** Sorafenib inhibits the interaction of GP73 and AFP. HepG2 cells co-transfected with HA-GP73 and FLAG-AFP with or without sorafenib (10 μmol/L, 24 h) treatment were lysed with IP lysis buffer and subjected to immunoprecipitation with anti-HA antibody followed by western blotting with anti-HA or anti-FLAG antibodies. **B** Sorafenib inhibits secretion levels of GP73 and AFP. The protein levels of GP73 and AFP in cell lysate and supernatant were measured by western blotting in HepG2 cells with or without sorafenib (10 μmol/L, 24 h) treatment. β-actin or GST was respectively used as an intracellular or extracellular loading control. **C**–**F** HepG2 and PLC cells were treated with DMSO, sorafenib, AFP + sorafenib, GP73 + sorafenib, and AFP + GP73 + sorafenib respectively. Cells were all treated with 10 μmol/L sorafenib for 24 h except cells in MTT study for 72 h. **C** Extracellular GP73 and AFP synergistically rescued sorafenib-induced expression of proliferation and metastasis-related proteins. The protein levels of PTEN, phospho-AKT, AKT, N-cadherin, E-cadherin, and MMP9 in the cells were measured by western blotting. β-actin was used as a control. **D** Extracellular GP73 and AFP synergistically resisted sorafenib-induced apoptosis. Cells were stained with Annexin V-FITC and PI, and cell apoptosis was analyzed by FACS. **E** Extracellular GP73 and AFP synergistically resisted anti-proliferation effect of sorafenib. Cell growth rates were measured by MTT assay. **F** Extracellular GP73 and AFP synergistically resisted anti-migration and anti-invasion effect of sorafenib. Migratory or invasive cells were stained with 0.1% crystal violet and observed by microscopy. **G** Extracellular AFP and GP73 synergistically resisted tumorigenesis inhibition of sorafenib. HepG2 cells were subcutaneously injected into nude mice. Sorafenib was given at a dose of 30 mg/kg/day orally. AFP or GP73 was given at a dose of 10 mg/kg via intraperitoneal injection twice a week. The tumors were weighed and measured for four weeks after injection. Error bars represent S.D. *p < 0.05, **p < 0.01. **H** Model of GP73-mediated secretion of AFP and GP73 and its impact on HCC progression. GP73 promotes AFP secretion through directly binding to AFP. GP73-mediated secretion of AFP and GP73 regulates expression of proliferation and metastasis-related proteins, thus promotes proliferation and metastasis of HCC cells synergistically. Sorafenib exerts its antitumor effect by inhibiting the interaction of GP73 and AFP, thereby reducing GP73-mediated secretion of AFP and GP73.

Sorafenib is the first FDA-approved first-line drug for treating advanced HCC [30]. Clinical studies show that high serum levels of AFP are associated with the resistance of HCC patients to therapy using sorafenib [31, 32]. We therefore next sought to investigate whether sorafenib affected GP73-mediated secretion. For this purpose, we co-transfected HepG2 cells with FLAG-AFP and HA-GP73 in the presence or absence of sorafenib. Coimmunoprecipitation assay using anti-FLAG or anti-HA antibodies showed that sorafenib treatment reduced the intracellular interactions of AFP and GP73 (Fig. 7A). Furthermore, sorafenib treatment decreased the levels of intracellular AFP and GP73 as well as those of secreted AFP and GP73 (Fig. 7B).

To determine whether extracellular AFP or GP73 influenced the antitumor effects of sorafenib, HepG2 and PLC cells were each treated with DMSO, sorafenib, AFP with sorafenib, GP73 with sorafenib, or AFP plus GP73 with sorafenib. We analyzed the levels of proliferation- and metastasis-related proteins and the phenotypes of the cells. As shown in Fig. 7C, sorafenib significantly reduced the levels of phosphorylated AKT, MMP9, and N-cadherin and increased those of PTEN and E-cadherin. Furthermore, treatment with AFP or GP73 with sorafenib moderately rescued the protein levels induced by sorafenib; and cotreatment with AFP and GP73 plus sorafenib highly rescued the levels of the target proteins induced by sorafenib.

We next performed proliferation, apoptosis, migration, and invasion assays of HepG2 and PLC cells subjected to the treatments described above. As shown in Fig. 7D, sorafenib significantly inhibited cell growth; whereas HepG2 and PLC cells showed modest proliferation following treatment with AFP or GP73 with sorafenib. Moreover, cotreatment with AFP and GP73 synergistically enhanced cell proliferation. Similarly, combining AFP and GP73 significantly inhibited sorafenib-induced apoptosis of HCC cells, and treatment with AFP or GP73 partially increased resistance to sorafenib (Fig. 7E). Furthermore, treatment with AFP or GP73 rescued migration and invasiveness, and the combination of AFP and GP73 exhibited a synergistic effect (Fig. 7F).

To investigate the biological functions of AFP and GP73 on the resistance of tumors to sorafenib, we used a nude mouse xenograft model of HCC. As shown in Fig. 7G, sorafenib significantly inhibited tumorigenesis, whereas treatment with AFP or GP73 promoted the tumorigenic behavior of HepG2 cells, and combination treatment synergized to promote tumorigenesis. Thus, these results indicate that extracellular AFP or GP73 reduced the cytotoxic effects of sorafenib, and when combined, HCC cells became more resistant to sorafenib.

## Discussion

HCC is one of the most common malignant tumors worldwide and is predominant in Asian countries. AFP and GP73 act as serum markers for the diagnosis of HCC [33], and clinical data reveal a possible correlation between the serum levels of GP73 and AFP [34]. Using bioinformatics analysis, we found a statistically significant positive correlation between GP73 and AFP expression in HCC patients from public databases (Fig. S6). Here, we investigated the effects of GP73-mediated AFP secretion on the malignant phenotype of HCC cells and characterized the underlying mechanisms (Fig. 7H). We found that GP73 interacted with AFP and promoted the secretion of AFP, thereby promoting the proliferation, migration, and invasiveness of HCC cells. Extracellular GP73 promoted the malignant phenotype of HCC cells independent of AFP. Moreover, extracellular AFP and GP73 synergized to enhance the proliferation, migration, and invasiveness of HCC cells through regulating the expression of proteins involved in proliferation and metastasis. Furthermore, defective GP73 secretion influenced the levels of secreted AFP to attenuate the malignant phenotype of HCC cells. When we investigated the influence of GP73 and AFP on the efficacy of the antitumor drug sorafenib, we found that extracellular GP73 or AFP inhibited the antitumor effects of sorafenib, such as increasing apoptosis and inhibiting proliferation, migration, and invasion. Notably, inhibition of these activities increased when HCC cells were co-treated with GP73 and AFP.

AFP regulates the phenotype of HCC cells through activation of the AKT and CXCR4 signaling pathways to promote cell growth and metastasis [14, 35]. However, the function of extracellular GP73 is unknown. Here we showed that extracellular GP73 promoted the proliferation, migration, and invasion of HCC cells through regulating the expression of AKT and metastasis-related proteins. GP73 exerted an oncogenic function in HCC cells in the presence or absence of AFP and AFPR expression, whereas extracellular AFP specifically affected HCC cells that expressed AFP and AFPR. These findings indicate that GP73 influences the malignant phenotype of HCC cells to a greater extent than AFP. These multiple mechanisms of GP73 functions in HCC cells require further study.

High serum levels of AFP are associated with a high recurrence rate and shorter survival of patients with HCC and serve as a marker for higher risk of HCC [36]. Furthermore, AFP plays an immunosuppressive role in the escape of HCC cells from the immune system in hepatocarcinogenesis [37, 38]. Moreover, high serum GP73 concentrations are associated with poor prognosis and shorter survival, and the serum levels of AFP and GP73 are widely used for screening, determining efficacy surveillance, and detecting recurrence [39, 40]. Moreover, extracellular AFP and GP73 influence the progression of HCC. For example, serum biomarkers of HCC such as AFP and glypican-3 serve as targets of HCC antibody therapy, which ongoing early-phase clinical trials indicate may be more efficacious than standard therapy [41]. Thus, GP73 and AFP may represent novel therapeutic targets for HCC.

AFP-positive HCC accounts for ~70–80% of cases worldwide. Our present study shows that extracellular GP73 affects AFP-negative HCC cells, and therefore GP73 may serve as a specific and sensitive target for AFP-negative HCC. Moreover, combination drug therapy for GP73 and AFP may achieve more effective treatment of AFP-positive HCC. In regard to the development of specific targeting drugs, our present study reveals the importance of the RVRR and TMD structures in mediating the secretion of GP73, which enhances the malignant phenotype of HCC cells. Therefore, the therapeutic potential of targeting these functional domains should be evaluated in detail.

Some studies reported that GP73 as a biomarker of HCC diagnosis is controversial, especially its significance in the assessment of tumor recurrence after hepatectomy remains obscure [20, 36, 42]. Kokudo et al. reported that AFP declined after hepatectomy of HCC patients with cirrhosis, however, the level of GP73 remained stable [36]. Given AFP is mainly produced by patients with nodular regeneration of the liver, whereas serum GP73 is elevated in patients with cirrhosis and HCC almost always arises in patients with cirrhosis, it is reasonable that AFP declined, but GP73 level did not change after hepatectomy of HCC patients with cirrhosis. Furthermore, the recurrence of elevated GP73 correlates with tumor recurrence [20, 42]. Maintaining elevated GP73 level indicates the existence of tumor lesions and thus may serve as an indicator for the recurrence of HCC. Therefore, GP73 is a valuable biomarker for HCC, and may also be used in the surveillance of HCC recurrence in postoperative management.

Patients are typically diagnosed with HCC during its intermediate or advanced stage, which presents a major obstacle to achieving treatment that improves prognosis. For example, sorafenib effectively treats advanced HCC. Our present findings that GP73 and AFP confer resistance to sorafenib may guide the development of novel therapeutic combinations and facilitate the selection of patients who will benefit from such therapy. Moreover, extracellular GP73 and AFP effectively confer resistance to the antitumor effects of sorafenib in vitro and in vivo. Therefore, pharmacological targeting of AFP and GP73 may improve the efficacy of antitumor drugs, thereby improving patients’ prognoses and prolonging their survival.

Together, our present results reveal that GP73 promotes the malignant phenotype of HCC cells by upregulating its own levels of secretion as well as those of AFP. Moreover, GP73-mediated secretion of AFP and GP73 plays an important role in the resistance of HCC cells to sorafenib, which provides a new perspective for further studies of the molecular mechanism of HCC progression. Thus, these findings contribute to a more comprehensive theoretical basis for optimizing the diagnosis and treatment of HCC as well as facilitating the identification of new targets of therapy.

## Materials and methods

### Cell culture, transfection, lentivirus infection, and CRISPR/Cas9

HEK293T, L02 (normal human liver cell), HepG2, PLC/PRF/5 and HLE (human HCC cell lines) were cultured in DMEM supplemented with 10% fetal bovine serum (FBS) at 37 °C in 5% CO_2_. Cell transfection were utilizing TurboFect transfection reagent (Thermo Scientific) referring to the manufacturer’s instruction. The transfection efficiency was confirmed after 48 h post transfection. The lentivirus plasmid PLVX-IRES was inserted with GP73, GP73^Mut^, GP73^TMD^ to acquire the stable expression in HepG2 and PLC cells. Cells were selected for one week with puromycin and tested the positive efficiency.

For the design of sgRNA in ZhangFeng library, sgRNA targets GP73 5′-GAGCGTCAACAAGCTGTACC-3′, which was inserted in CRISPR vector pSpCas9(BB)−2A-GFP. HepG2 and PLC cells were transfected with the expression plasmid and were separated by Flow Cytometer to single positive cell. Cells were cultured for one month and detected the effects of knockout expression.

### Reagents

Sorafenib was purchased from LC labs, Rapamycin was purchased from MedChemExpress. Antibodies against HA (MMS101P, Covance), FLAG (F1804, Sigma), GST (IT003 M, M&C Gene Technology), His (66005-1-Ig, Proteintech), TGN46 (13573-1-AP Proteintech), AFP (4550-1-AP, Proteintech), GP73(sc-365817, Santa Cruz), E-cadherin (sc-8426, Santa Cruz), N-cadherin (22018-1-AP, Proteintech), MMP9 (10375-2-AP, Proteintech), PTEN (9188, CST), p-AKT (4060, CST), AKT (9272, CST), β-actin (AC026, ABclonal).

### Western blotting

Cells were collected and lysed in RIPA buffer (Thermo Scientific) including protease inhibitor cocktail (Sigma) and protein concentrations were measured utilizing the BCA protein assay kit (Pierce). A total of 25–40 μg of protein was separated by SDS-PAGE and transferred to nitrocellulose membranes (Pall). The following primary antibodies for western blotting were in reagents. The fluorescence labeled secondary antibodies we used were as follows: anti-mouse IgG antibody DyLight 800 (610-145-121, Rockland) and anti-rabbit IgG DyLight 800 (611-145-002, Rockland). The infrared fluorescence graphic results were acquired utilizing Odyssey infrared imaging system (LICOR Bioscience, Lincoln, NE).

### Real-time PCR

Total RNA extraction was utilizing the RNAsimple Total RNA kit (Tiangen). Reverse transcription was performed utilizing ReverAid FirstStrand cDNA Synthesis kit (Vazyme). The mRNA expression level was detected by real-time PCR utilizing Maxima SYBR Green qPCR Master Mix (Vazyme). The primers’ sequences were shown as follows: human AFP primers: 5′-CCAACAGGAGGCCATGCTT-3′ and 5′-GAATGCAGGAGGGACATATGTTT-3′; GAPDH primers: 5′-CCATGGAGAAGGC-TGGGG-3′ and 5′-CAAAGTTGTCATGGATGACC-3′. The mRNA expression was normalized by GAPDH.

### Immunoprecipitation

Cells were collected and lysed with IP lysis buffer (25 mM Tris-HCl (pH 7.4), 150 mM NaCl, 1% Nonidet P-40, 1 mM EDTA, and 5% glycerol) including protease inhibitor cocktail (Sigma) for one hour at 4 °C. The samples were incubated with Protein A-Sepharose (GE Healthcare) and the indicated antibody on a rocking platform overnight at 4 °C. The next day, the beads were washed with IP lysis buffer three times, boiled in 2× SDS loading buffer for 10 min at 95 °C and subsequently detected by western blotting with indicated antibodies.

### Immunofluorescence assay

Transfected cells were cultured in 3.5 cm confocal dishes and washed with PBS. Then, cells were fixed with 4% paraformaldehyde for 15 min and permeabilized (using 0.2% Triton X-100 in PBS) for 15 min under room temperature. After washing with PBS for three times, cells were blocked for 1 h with 1% bovine serum albumin in PBS under room temperature and incubated with primary antibodies overnight at 4 °C. The next day, cells were incubated with the secondary antibodies conjugated with AlexaFlour488 (anti-rabbit IgG) and AlexaFlour549 (anti-mouse IgG). DNA was stained with DAPI at final concentration of 1 μg/ml for 1 min. Images were photographed using a ZEISS fluorescence microscope.

### GST pull-down assay

The control GST and GST-tagged proteins were expressed in Escherichia coli strain BL21 (DE3). Bacterial lysates were prepared in ice-cold binding buffer (PBS) by sonication and incubated with glutathione-Sepharose beads (GE Healthcare) overnight at 4 °C with rocking. After the incubation, His-tagged proteins were added to each tube for 4 h at 4 °C. The beads were washed three times, and boiled in SDS-PAGE loading buffer and analyzed by western blotting with the indicated antibodies.

### Enzyme-linked immunosorbent assay (ELISA)

For quantitate AFP from cell culture supernatant use Human AFP ELISA Kit (ab193765, Abcam). The detail was described in the protocol of this kit.

### 3-(4,5-dimethylthyazol-2-yl)−2,5-diphenyltetrazolium bromide (MTT) assay

Cells were plated in 96-well plates at a density of 2000 cells/well. Cells were treated with different cell culture supernatant. After treating for 0, 12, 24, 36, 48, 60, 72 h, 15 μl of MTT solution (5 mg/ml) was added to each well, followed by further incubation at 37 °C for 4 h. The medium was removed and 200 μl DMSO was added to each well to dissolve the formazan crystals. The absorbance at 490 nm was read using the microplate reader. The MTT assay was performed on three biological replicates, and each replicate was measured at least three times.

### Cell migration and invasion assay

For the cell migration assay, about 5 × 10^4^ HepG2, HLE, or PLC cells were added into the upper chambers of the wells and cultured with serum-free DMEM medium, whereas stable expressing cells were plated in the lower chambers and cultured with DMEM medium containing 10% FCS. After 24 h incubation at 37 °C, the migrated cells were fixed with 4% paraformaldehyde and stained by 0.1% crystal violet. Then the cells were photographed by microscopy (Leica) and counted by image J software. For the invasion assay, the upper chambers were covered with Matrigel. Then the following experimental procedure is similar to the migration.

### Cell apoptosis assay

The cell apoptosis was evaluated using the Annexin V-FITC/propidium iodide (PI) Apoptosis Detection Kit (KGA108, KeyGEN BioTECH). HepG2 and PLC cells were treated with DMSO, sorafenib, AFP + sorafenib, or GP73 + sorafenib, or AFP + GP73 + sorafenib for 24 h. After harvest, the cells were digested with trypsin without EDTA, washed twice with PBS and resuspended in 500 μl binding buffer. Then the cells were incubated with 5 μl Annexin V-FITC for 15 min and 5 μl PI for another 5 min at room temperature in the dark. After staining, cells were analyzed by a flow cytometer (BD Biosciences).

### Tumorigenicity in nude mice

HepG2 cells (5 × 10^6^/0.2 mL) were subcutaneously injected into five-week-old male nude mice with left or right hind legs. After tumor establishment, mice were randomly assigned to treatment with vehicle, sorafenib (30 mg/kg/day), AFP (10 mg/kg, twice a week) with sorafenib (30 mg/kg/d), GP73 (10 mg/kg, twice a week) with sorafenib (30 mg/kg/d), and AFP (10 mg/kg, twice a week), GP73 (10 mg/kg, twice a week) with sorafenib (30 mg/kg/d). After injection for 4 weeks, the mice were killed, and the tumors were weighed and measured. All experiments and facilities were approved by the Committee for Ethics of Animal Experiments and were conducted in conformity to the Guidelines for Animal Experiments, Peking University Health Science Center.

### Statistical analysis

Statistical analyses were performed using SPSS 22.0 software. Data were presented as mean ± S.D. (standard error of mean) from at least three-independent experiments. The statistical analysis was done by using Student’s *t* test, and *p* < 0.05 was considered statistically significant, *p < 0.05, **p < 0.01.

## Supplementary information

Supplementary figure legends

Figure S1

Figure S2

Figure S3

Figure S4

Figure S5

Figure S6
