# Supplementary material for: GP73-mediated secretion of AFP and GP73 promotes proliferation and metastasis of hepatocellular carcinoma cells
Source: Oncogenesis. 2021 Oct 14;10(10):69. doi: 10.1038/s41389-021-00358-3 (PMC8516944; doi:10.1038/s41389-021-00358-3)
Supplement: Supplementary file 1 — Supplementary figure legends [file 41389_2021_358_MOESM1_ESM.docx]

Supplementary figure legends:

Figure S1. GP73-mediated secretion has no effect on proliferation in L02 cells. L02 cells treated with or without GP73-OE SP were analyzed by MTT assay.

Figure S2. GP73 may bind to a specific receptor on the cell surface. HepG2 cells were co-cultured with or without FITC tagged-GP73.

Figure S3. GP73 binds to AFP in supernatant. Cell culture supernatants from HepG2 cells that stably overexpressed GP73 were collected and subjected to immunoprecipitation with AFP antibody followed by western blotting with GP73 antibody.

Figure S4. GP73-induced proliferation and metastasis of HCC cells requires AFP binding domain. MTT (A) and Transwell assays (B) were conducted with HepG2 with treatment of vector, GP73, Δ56-92, Δ91-150, or Δ146-205 overexpression supernatant. Scale bar, 200 μm. Error bars represent S.D. *, p < 0.05, **, p<0.01. NS, no significance.

Figure S5. Rapamycin inhibits malignancy of HCC cells through reducing the binding of GP73 and AFP. (A) Rapamycin inhibits the interaction of GP73 and AFP. HepG2 cells co-transfected with HA-GP73 and FLAG-AFP with or without rapamycin (20 nmol/L, 24 h) treatment were conducted with coimmunoprecipitation assay. (B) Rapamycin inhibits GP73 secretion level. The protein levels of GP73 in cell lysate and supernatant were measured by western blotting in HepG2 cells with or without rapamycin (20 nmol/L, 24 h) treatment. β-actin or GST was respectively used as an intracellular or extracellular loading control. (C) Rapamycin inhibits expression of proliferation and metastasis-related proteins. The protein levels of GP73, phospho-AKT, AKT, N-cadherin, E-cadherin and MMP9 in HepG2 cells with or without rapamycin (20 nmol/L, 24 h) treatment were measured by western blotting. β-actin was used as a control. (D)-(E) Rapamycin inhibits malignancy of HepG2 cells. MTT (D) and Transwell assays (B) were conducted with HepG2 with or without rapamycin (20 nmol/L, 24 h) treatment. Scale bar, 200 μm. Error bars represent S.D. **, p<0.01.

Figure S6. GP73 expression positively correlates with AFP expression in HCC patients. The data is derived from public database: <https://www.proteinatlas.org/>. The linear regression and correlation were analyzed by GraphPad Prism 5.
