# Supplementary figures and images for: GP73-mediated secretion of AFP and GP73 promotes proliferation and metastasis of hepatocellular carcinoma cells
Source: Oncogenesis. 2021 Oct 14;10(10):69. doi: 10.1038/s41389-021-00358-3 (PMC8516944; doi:10.1038/s41389-021-00358-3)

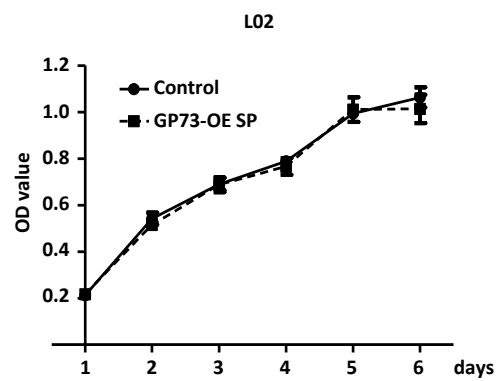

Figure S1

Supplement: Supplementary file 2 — Figure S1 [file 41389_2021_358_MOESM2_ESM.pdf]

FITC-GST-GP73

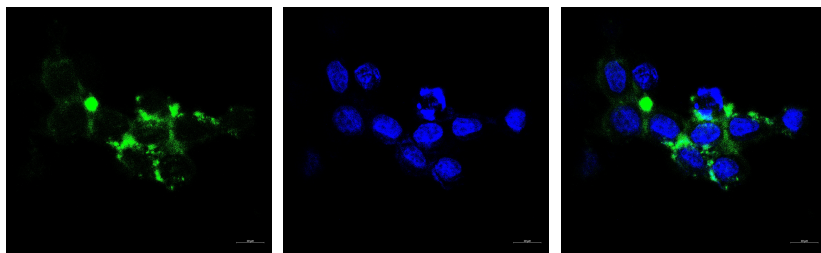

FITC-GST

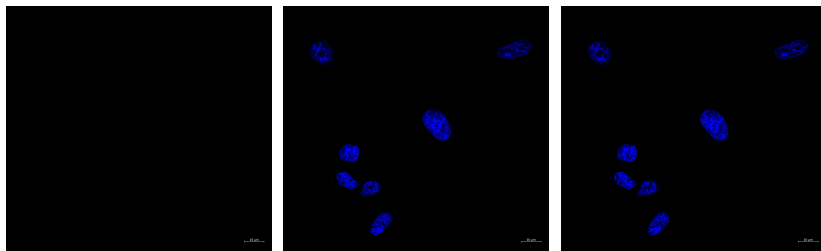

FITC

DAPI

Merge

Supplement: Supplementary file 3 — Figure S2 [file 41389_2021_358_MOESM3_ESM.pdf]

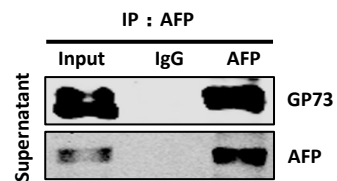

Figure S3

Supplement: Supplementary file 4 — Figure S3 [file 41389_2021_358_MOESM4_ESM.pdf]

**A**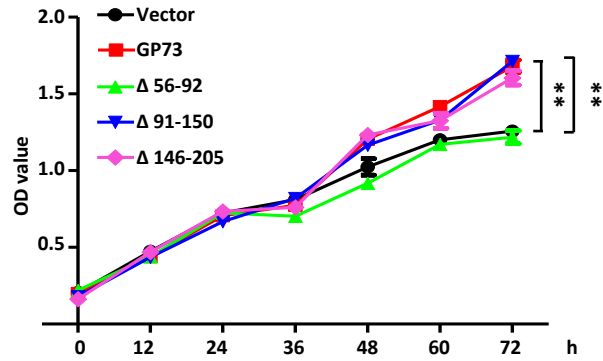**B**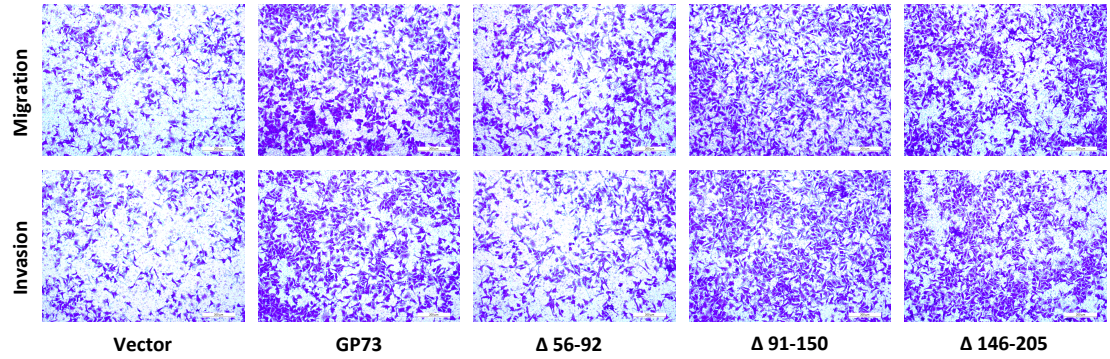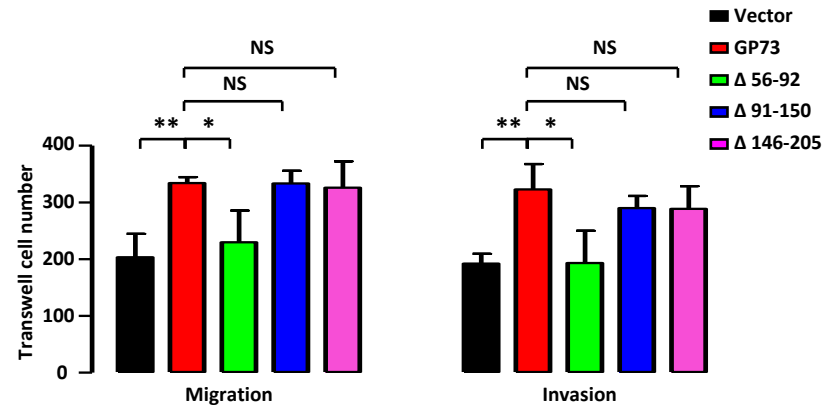**Figure S4**

Supplement: Supplementary file 5 — Figure S4 [file 41389_2021_358_MOESM5_ESM.pdf]

**A**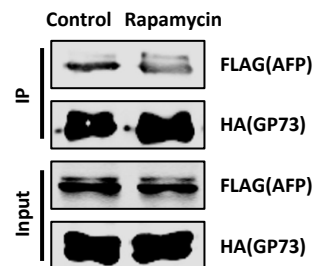**B**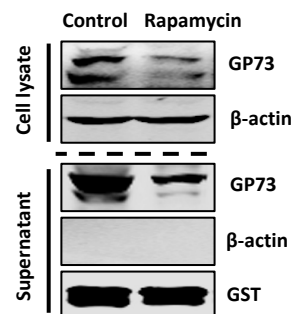**C**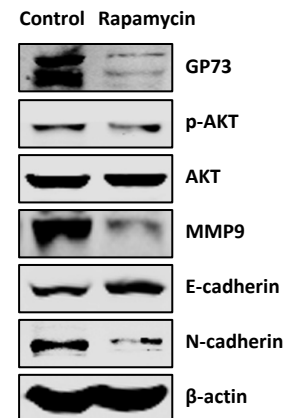**D**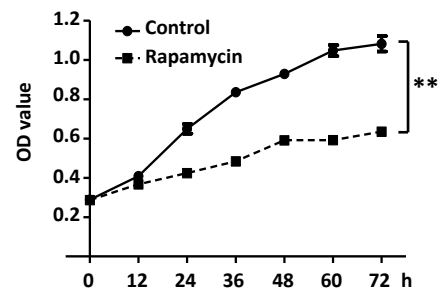**E**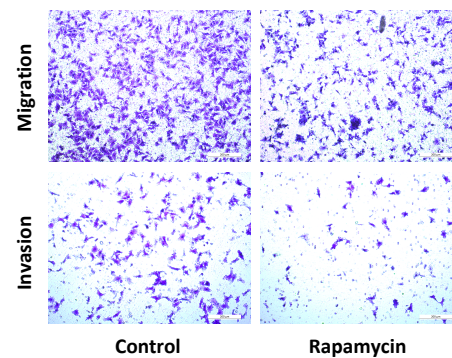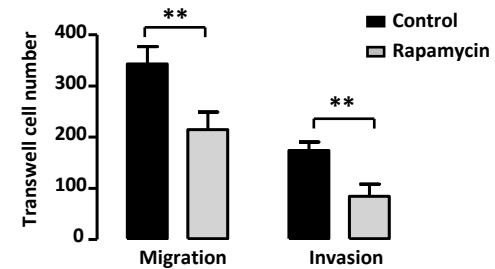**Figure S5**

Supplement: Supplementary file 6 — Figure S5 [file 41389_2021_358_MOESM6_ESM.pdf]

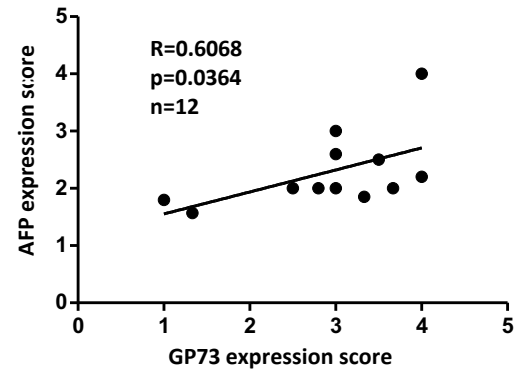

<https://www.proteinatlas.org/>

Supplement: Supplementary file 7 — Figure S6 [file 41389_2021_358_MOESM7_ESM.pdf]
